# The clinicopathological significance of Mortalin overexpression in invasive ductal carcinoma of breast

**DOI:** 10.1186/s13046-016-0316-0

**Published:** 2016-03-09

**Authors:** Haidan Jin, Meiying Ji, Liyan Chen, Qixiang Liu, Shuanlong Che, Ming Xu, Zhenhua Lin

**Affiliations:** Department of Pathology & Cancer Research Center, Yanbian University Medical College, Yanji, 133002 China; Department of Biochemistry, Yanbian University Medical College, Yanji, 133002 China; Department of Breast Surgery, The Second Hospital of Jilin University, Changchun, 130041 China

**Keywords:** Breast cancer, Mortalin, Survival analysis, Biomarker

## Abstract

**Background:**

Mortalin/GRP75 is a ubiquitous mitochondrial chaperone which related to the cytosolic heat shock protein 70 (HSP70), and plays a role in carcinogenesis. This study aims to investigate the Mortalin expression in breast cancer and its correlation with the outcome of the patients with breast cancer.

**Methods:**

A total of 155 invasive ductal carcinoma of breast patients with strict follow-up, 52 ductal carcinoma in situ (DCIS) and 45 adjacent non-tumor breast tissues were selected for immunohistochemical (IHC) staining of Mortalin protein. The localization of Mortalin protein was detected in MDA-MB231 breast cancer cells using immunofluorescence (IF) staining. The correlations between overexpression of Mortalin and the clinical features of patients with breast cancer were evaluated using chi-square test and Fisher’s exact tests. The survival rates were calculated by the Kaplan-Meier method, and the relationship between prognostic factors and patient survival was also analyzed by the Cox proportional hazard models.

**Results:**

Mortalin protein showed a mainly cytoplasmic staining pattern in breast cancers by using IHC staining in paraffin embedded breast cancer tissues and IF staining in MDA-MB231 breast cancer cells. The strongly positive rate of Mortalin protein was 63.9 % (99/155) in invasive ductal carcinoma of breast and was significantly higher than in DCIS 34.6 % (18/52) and adjacent non-tumor tissues 15.6 % (7/45). Overexpression of Mortalin was closely correlated with histological grade, clinical stage, lymph node metastasis, lower disease free survival (DFS) and overall survival (OS) rates of patients with breast cancer. Moreover, multivariate analysis suggested that Mortalin emerged as a significant independent prognostic factor along with clinical stage and Her2 expression status in patients with breast cancer.

**Conclusions:**

Mortalin is upregulated in breast cancer, and may be a useful poor prognostic biomarker as well as a potential therapeutic target for patients with breast cancer.

## Background

Breast cancer, which is the most prevalent malignancy in women worldwide, is of growing concern because of its rising incidence and ongoing regional disparities in incidence [1]. In eastern China, which accounts for 38 % of the Chinese population, there has been a sharp increase in breast cancer incidence in recent decades [2]. Even though there has been great improvement on traditional treatments, such as surgery, supplemented with chemotherapy, a large number of patients with breast cancer are diagnosed at the advanced stages and the prognosis of these patients are still poor. In this context, biomarker-based assays and molecular methods will offer added values. Mortalin is a highly conserved molecular chaperone in the heat shock protein (HSP) 70 family, which is encoded by the nuclear gene HSPA9B (GeneID: 3313) localized on chromosome 5q31.1.1. [3]. It plays an important role in human carcinogenesis by enhancing cancer cell proliferation, protecting cancer cells against apoptosis and promoting cancer angiogenesis [4, 5]. Overexpression of Mortalin may also interacts with the wild-type tumor suppressor protein p53 and modulates the Ras-Raf-MAPK pathway and then increase the malignancy of tumor cells [6–8]. Wadhwa et al. have shown that Mortalin is elevated in human brain tumor, colon carcinoma, leukemia, and the immortalized cell lines derived from the tumors [4]. It is associated with positive venous infiltration and advanced tumor TNM stages in Hepatocellular carcinoma [9]. Starenki et al. found that Mortalin is upregulated in human medullary thyroid carcinoma tissues and its depletion robustly induces cell death and growth arrest in medullary thyroid carcinoma cell lines and mouse xenografts [10].

Here we performed immunohistochemical (IHC) staining of Mortalin protein in 252 cases of invasive ductal carcinoma of breast, DCIS and adjacent non-tumor tissues, and found that Mortalin protein was frequently overexpressed in breast cancer compared with the adjacent non-tumor tissues. The overexpression of Mortalin in breast cancer was associated with histological grade, clinical stage and lymph node metastasis. Multivariate analysis revealed that Mortalin might be an independent biomarker for the prediction of breast cancer prognosis.

## Methods

### Ethics statement

This study was in accordance with the Declaration of Helsinki and approved by the Human Ethics committee and the Research Ethics committees of Jilin University Medical College in China. Patients were fully informed that the resected specimens were kept by the hospital and might be used for scientific research, and their privacy would be maintained. Follow-up survival data were collected retrospectively through medical record analyses.

### Clinical samples

A total of 155 invasive ductal carcinoma of breast tissue samples were used in this study. All samples were routinely fixed in 10 % buffered formalin and embedded in paraffin blocks. The study protocol was approved by the institutional review board of Jilin University Medical College. The pathological parameters, including age, histological grade, clinical stage, nodal metastasis and survival data, were carefully reviewed in all 155 invasive ductal carcinoma of breast. The patients’ ages ranged from 37 to 72 years with a mean age of 47.7 years. For grading of invasive ductal carcinoma of breast, 62 cases were G1, 48 cases were G2, and 45 cases were G3. For staging of invasive ductal carcinoma of breast, 92 cases were TNM stages 0–II, and 63 cases were TNM stages III–IV. TNM staging was assessed according to the staging system established by the American Joint Committee on Cancer [11].

### Immunofluorescence (IF) staining analysis

Breast cancer MDA-MB231 cells were grown on coverslips to 70 % confluence. The cells were fixed with 4 % paraformaldehyde for 10 min, and after 24 h cells were permeabilized with 0.5 % TritonX-100 for 10 min. Blocking was performed with 3 % Albumin Bovine V (A8020, Solarbio, Beijing, China) for 1 h at room temperature. After washing with PBS, cells were incubated with rabbit anti-Mortalin antibody (ab53098) (Abcam, Cambridge, MA, USA), at 4 °C overnight, followed by incubation with Alexa Fluor 488 Goat Anti-Rabbit IgG (H + C) (A11008, 1:1000, Invitrogen, USA) for 1 h at room temperature. After washing with PBS, the cells were counterstained with DAPI (C1006, Beyotime, Shanghai, China), and the coverslips were mounted with an Anti fade Mounting Medium (P0126, Beyotime, Shanghai, China). IF signals were visualized and recorded with a BX53 Olympus microscope.

### Immunohistochemical (IHC) staining analysis

The Dako LSAB kit (Dako, Glostrup, Denmark) was used for IHC. Serial 4 μm-thick tissue sections were prepared on silane-coated slides (Sigma, St. Louis, MO, USA), and deparaffinized, rehydrated and incubated with 3 % H_2_O_2_ in methanol for 10 min at room temperature to eliminate endogenous peroxidase activity. The antigen was retrieved at 95 °C for 20 min by placing the slides in 0.01 M sodium citrate buffer (pH 6.0). Slides were then incubated with the primary antibody of rabbit anti-Mortalin antibody (ab53098) (Abcam, Cambridge, MA, USA), at 4 °C overnight. After incubation at room temperature for 30 min with biotinylated secondary antibody, slides were incubated with streptavidin–peroxidase complex at room temperature for 30 min. Slides were immunostained with 3,3’-diaminobenzidine chromogen and then counterstained with Mayer’s hematoxylin. We used tonsil sections as the positive control and Rabbit IgG as an isotope control. In addition, tissue sections were processed omitting the primary antibody as the negative control.

### Analysis of IHC results

All slides were scored independently by two pathologists (Lin Z and Jin H) who were blind to all clinical data. In case of discrepancies, a final score was established by reassessment of both pathologists on a double-headed microscope. Interpretation criteria were as previously described [12]. Cytoplasmic expression patterns were considered as positive staining. Staining intensity of tissue sections scored as ‘–’ no staining, ‘+’ definite but weak staining, ‘++’ and ‘+++’ were considered as moderate and intense staining of Mortalin. The staining area was scored as follows: scored as ‘−’ (negative, no or less than 5 % positive cells), ‘+’ (5–25 % positive cells), ‘++’ (26–50 % positive cells) and ‘+++’ (more than 50 % positive cells). Taking the double scoring system together, ‘++’ or ‘+++’ scored samples were considered as high Mortalin expression, and ‘−’or ‘+’scored samples were considered as low Mortalin expression.

### Statistical analysis

Statistical analysis was performed using SPSS version 17.0 software for Windows (SPSS, Chicago, IL, USA). Correlations between Mortalin expression and clinicopathological characteristics were evaluated using Chi-square tests (*χ2*) and Fisher's exact tests. Survival rates were calculated using the Kaplan-Meier method, and differences in survival curves were analyzed by log-rank tests. Univariate and multivariate survival analyses were performed on all characteristics using the Cox proportional hazard regression model. A *P*-value less than 0.05 was considered statistically significant.

## Results

### Mortalin protein expression in invasive ductal carcinoma and normal epithelia of breast

**Fig 1:**
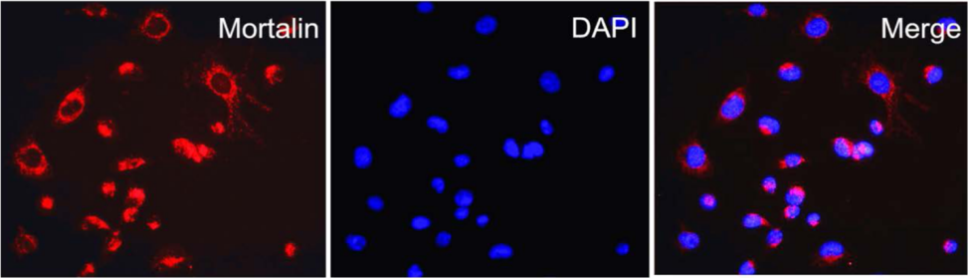
Immunofluorescence staining for Mortalin protein in MDA-MB231 breast cancer cells. MDA-MB231 breast cancer cells were immunostained for Mortalin (red). Nuclei were visualized by DAPI staining (blue). Mortalin protein is mainly located in the cytoplasm of MDA-MB231 cancer cells

**Fig 2:**
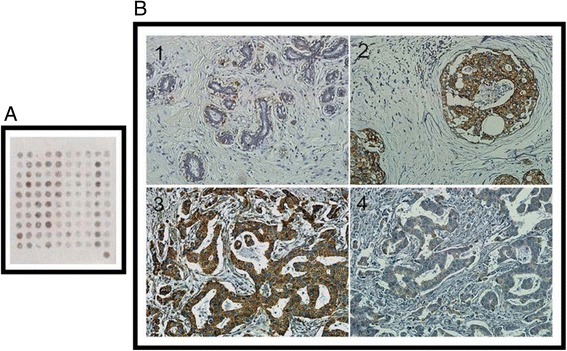
**a**. Mortalin protein was detected in tissue microarray of breast lesions by Immunohistochemical staining. **b**1. Normal breast tissues are negative for Mortalin expression. **b**2. Positive Mortalin protein expression was detected in the cytoplasm of DCIS. **b**3. Mortalin protein is strongly positive in breast cancer with lymph node metastasis. **b4**. Mortalin protein is weak positive in breast cancer without metastasis. **b1**:×100; **b2**-**b4**:×200

IF staining revealed strongly positive signals for Mortalin protein in the cytoplasm of MDA-MB231 breast cancer cells (Fig. 1). IHC staining demonstrated that Motalin protein was mainly located in the cytoplasm of breast cancers. The positive rate of Mortalin protein was significantly higher in breast cancers (78.1 %, 121/155) than either in DCIS (53.8 %, 28/52) or in adjacent normal breast tissues (28.9 %, 13/45) (*P* < 0.01 and *P* < 0.05, respectively). Similarly, the strongly positive rate of Mortalin protein was higher in both breast cancers (63.9 %, 99/155) and DCIS (34.6 %, 18/52) compared with normal breast tissue (15.6 %, 7/45). Interestingly, positive Mortalin protein expression was also frequently observed in blood vessels and/or lymphatic vessels in the stroma of breast cancer and the adjacent non-tumor tissues (Fig. 2, Table 1).

**Table 1:** Mortalin protein expression in breast cancers

Diagnosis	No. of cases	Positive cases	Positive cases rates	Strongly positive rates
		- + ++ +++		
Invasive ductal carcinoma of breast	155	34 22 53 46	78.1 %**	63.9 %**
DCIS	52	24 10 12 6	53.8 %*	34.6 %*
Adjacent non-tumor	45	32 6 7 0	28.9 %	15.6 %

*DCIS*; ductal carcinoma *in situ*

Positive rate: percentage of positive cases with +, ++, and +++ staining score. Strongly positive rate: (high-level expression) percentage of positive cases with ++ and +++ staining score

* *p* < 0.05 and ** *p* < 0.01 compared with non-tumor tissues

### Correlations between Mortalin protein overexpression and clinical parameters of breast cancer

**Fig 3:**
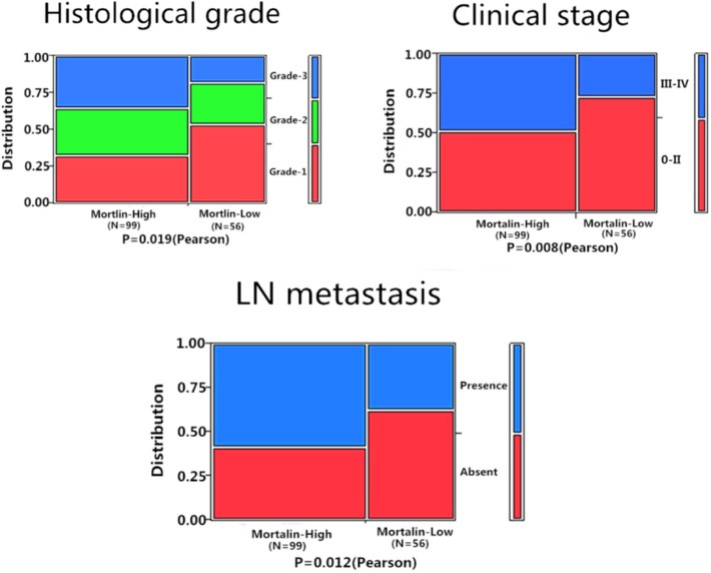
Relationship between Mortalin expression and clinicopathological significance of breast cancer. The expression level of Mortalin protein was significantly related with Histological grade (*P* = 0.019), Clinical stage (*P* = 0.008) and LN metastasis (*P* = 0.012)

Overexpression of Mortalin protein was significantly correlated with histological grade, clinical stage, and lymph node metastasis of breast cancer. However, Mortalin protein expression was not related with patient age, menopausal status, ER or PR levels and Her2 status in breast cancer. For histological grade, the strongly positive rate of Mortalin protein was significantly higher in Grade-3 breast cancers (77.8 %, 35/45) than in Grade-2 (66.7 %, 32/48) and Grade-1 (51.6 %, 32/62). The strongly positive rate of Mortalin protein was 76.2 % (48/63) in breast cancers with clinical stage III-IV, which was significantly higher than in cases with clinical stage 0-II (55.4 %, 51/92). Similarly, the strongly positive rate of Mortalin protein was higher in breast cancers with lymph node metastasis (73.4 %, 58/79) compared with those with no metastasis (53.9 %, 41/76). Taken as a whole, the expression of Mortalin protein was positively correlated with histological grade, clinical stage and lymph node metastasis (Table 2, Fig. 3).

**Table 2:** Correlation between Mortalin protein expression and the clinicopathological parameters of breast cancer

Variables	No. of cases	Mortalin strongly positive cases (%)	*χ* ^*2*^	*P* value
Age			0.044	0.834
≥50	82	53 (64.6 %)		
<50	73	46 (63.0 %)		
Menopausal status			0.839	0.361
premenopausal	74	50 (67.6 %)		
Postmenopausal	81	49 (60.5 %)		
Histological grade			7.971	0.019**
Grade-1	62	32 (51.6 %)		
Grade-2	48	32 (66.7 %)		
Grade-3	45	35 (77.8 %)		
Clinical stage			6.981	0.008**
0-II	92	51 (55.4 %)		
III-IV	63	48 (76.2 %)		
LN metastasis			6.364	0.012**
Absent	76	41 (53.9 %)		
Presence	79	58 (73.4 %)		
ER			1.082	0.300
Positive	94	57 (60.6 %)		
Negative	61	42 (68.8 %)		
PR			0.926	0.338
Positive	89	54 (60.7 %)		
Negative	66	45 (68.2 %)		
Her2 status			1.609	0.206
Positive	96	65 (67.7 %)		
Negative	59	34 (57.6 %)		

* *p* < 0.05 and ** *p* < 0.01

### Association between overexpression of Mortalin and the prognosis of patients with breast cancer

**Fig 4:**
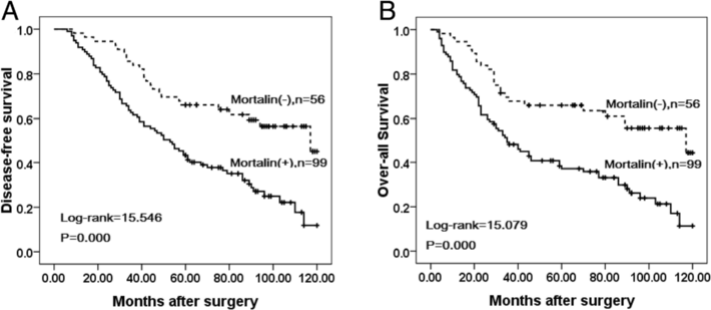
Kaplan–Meier analyses of disease-free and over-all survival rates in 155 breast cancer patients in relation to Mortalin protein overexpression. Breast cancer Patients with high Mortalin expression had (**a**) lower disease-free (*P* = 0.000) and (**b**) over-all (*P* = 0.000) survival rates than those with low Mortalin expression as determined using the Kaplan–Meier method

**Fig 5:**
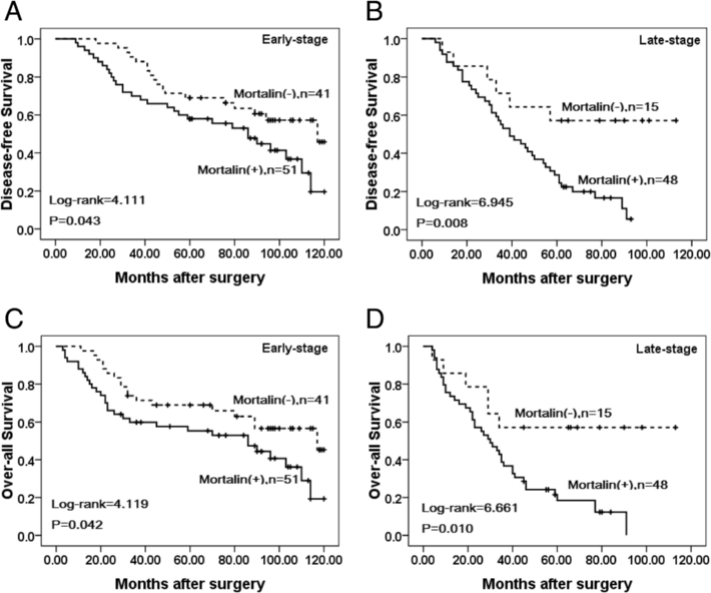
Kaplan–Meier survival curves of breast cancer patients in early and late stage. **a** and **c** show comparison of DFS and OS, respectively, in Mortalin (L) and (H) patients of early stage. **b** and **d** show comparison of DFS and OS, respectively in Mortalin (L) and (H) patients of late stage

**Fig 6:**
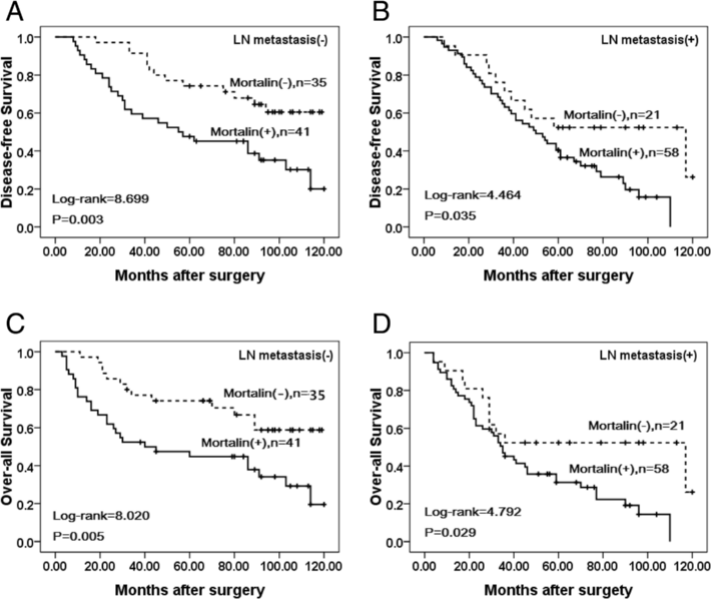
Kaplan–Meier survival curves of breast cancers patients with lymph node metastasis and without metastasis. **a** and **c** show comparison of DFS and OS, respectively, in Mortalin (L) and (H) patients without lymph node metastasis. **b**and **d** show comparison of DFS and OS, respectively in Mortalin (L) and (H) patients with lymph node metastasis

**Fig 7:**
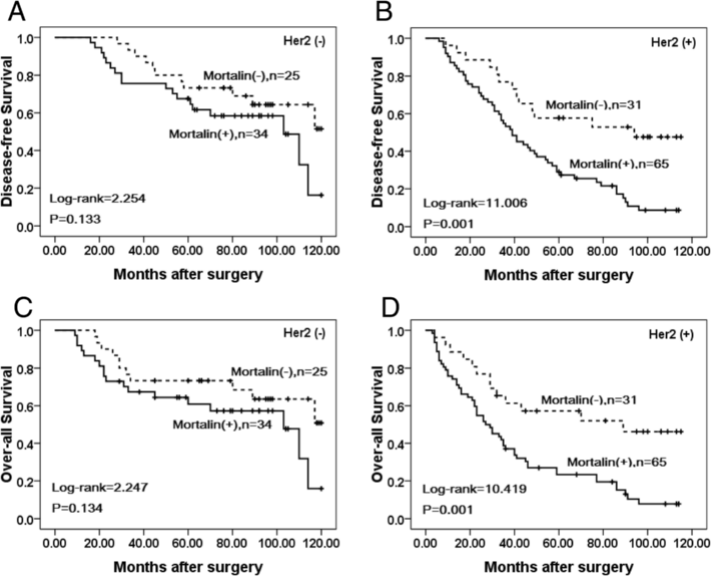
Kaplan–Meier survival curves of breast cancers with high Her2 level and low Her2 level patients. **a** and **c** show comparison of DFS and OS, respectively, in Mortalin (L) and (H) patients with low Her2 level. **b** and **d** show comparison of DFS and OS, respectively in Mortalin (L) and (H) patients with high Her2 level

Univariate analysis demonstrated that Clinical stage (*P* = 0.000), LN metastasis (*P* =0.006), Her2 expression levels (*P* = 0.001), and Mortalin expression status (*P* = 0.001) were significantly associated with DFS and OS in patients with breast cancer (Table 3). These data suggested that Mortalin could be a valuable prognostic factor in breast cancer. Further multivariate analysis using the Cox proportional hazards model revealed that Mortalin overexpression emerged as a significant independent prognostic factor for survival along with clinical stage and Her2 expression in breast cancer (*P* = 0.015). To further substantiate the importance of high Mortalin expression in breast cancer progression, we analyzed DFS and OS of 155 breast cancer cases using the Kaplan-Meier method, and found that patients with high Mortalin expression had lower DFS and OS than those with low Mortalin expression (both *P* < 0.0001) (Fig. 4). Additionally, breast cancer patients with high Mortalin expression had decreased DFS and OS compared to those with low Mortalin expression in either Early stage cases (*P* = 0.043, *P* = 0.042 respectively) or late-stage cases (*P* = 0.008, *P* = 0.010 respectively) (Fig. 5). Similarly, the patients with high Mortalin expression had decreased DFS and OS compared to those with low Mortalin expression in either LN metastasis (−) cases (*P* = 0.003, *P* = 0.005 respectively) or LN metastasis (+) cases (*P* = 0.035, *P* = 0.029 respectively) (Fig. 6). However, in the groups of patients with Her2 (−) cases, DFS and OS rate were not correlated with Mortalin expression status (*P* = 0.133, *P* = 0.134 respectively) (Fig. 7).

**Table 3:** Univariate and multivariate survival analyses (Cox regression model) of various factors in 155 breast cancer patients

Characteristics	B	SE	Wald	HR	95%CI		*P* value
					Lower	Upper	
Univariate survival analyses							
Age	0.212	0.162	1.724	1.237	0.901	1.698	0.189
Menopausal status	0.285	0.165	2.982	1.330	0.962	1.838	0.084
Histological grade	0.049	0.098	0.256	1.051	0.868	1.272	0.613
Clinical stage	0.870	0.179	23.608	2.386	1.680	3.389	0.000**
LN metastasis	0.451	0.164	7.521	1.570	1.137	2.166	0.006**
ER	0.241	0.164	2.164	1.273	0.923	1.755	0.141
PR	0.094	0.164	0.326	1.098	0.796	1.514	0.568
Her2	0.579	0.169	11.789	1.785	1.282	2.484	0.001**
Mortalin	0.550	0.171	10.343	1.732	1.239	2.422	0.001**
Multivariate survival analyses							
Clinical stage	0.690	0.187	13.615	1.994	1.382	2.876	0.000**
LN metastasis	0.270	0.170	2.518	1.310	0.938	1.829	0.113
Her2	0.442	0.172	6.575	1.556	1.110	2.182	0.010*
Mortalin	0.427	0.175	5.940	1.533	1.087	2.162	0.015*

* *p* < 0.05 and ** *p* < 0.01

## Discussion

In the current investigation, we focused on Mortalin/mthsp70/GRP75, a member of the heat shock protein (Hsp) 70 family, which is enriched in human cancer cells [4, 9, 13]. It has been established that Mortalin has various subcellular localizations, interacts with multiple binding partners, and plays a role in carcinogenesis. Also, it is elevated in immortalized cell lines and tumor cells, and additional upregulation of Mortalin expression at later stages of carcinogenesis coincides with the acquisition of invasiveness [4]. Moreover, it has multiple functions contributing to continued proliferation of cancer cells, including mitochondrial-biogenesis, ATP production, anti-apoptosis, chaperoning, inactivation of tumor suppressor p53 and PI3K/AKT activities [14, 15]. Targeting Mortalin by siRNA, ribozymes and small molecules including MKT-077 and Withaferin A resulted in growth arrest/apoptosis of cancer cells [16–22].

Here, we confirmed that the level of Mortalin was elevated in breast cancer, which supports the premise of overexpression of Mortalin in promoting human carcinogenesis. Compared with adjacent non-tumor tissues, Mortalin protein was found to be significantly up-regulated in breast cancer using IHC. Shin et al. stated that an overabundance of Mortalin expression on the cell surface was found by comparative proteome profiling of the cell surface and plasma membrane proteomes of various cancer and normal cells [23]. We also found that Mortalin protein was mainly localized in the cytoplasm of breast cancer cells using IF staining in MDA-MB231 cancer cells and IHC analysis of paraffin-embedded breast cancer tissues. Moreover, our IHC results showed that the positive rate of Mortalin protein in DCIS was also significantly higher than adjacent normal tissues, indicating that over expression of Mortalin may occur in the initiation stage of breast cancer progression. Recent studies have shown that the expression levels of Mortalin in cell lines with higher metastatic potential were significantly higher compared to those with lower metastatic potential. Compared with paracarcinomatous tissues and normal liver tissues, the expression of Mortalin was significantly increased in Hepatocellular carcinoma tumor tissues [24]. The present study clearly indicates that positive Mortalin protein expression was also observed in blood vessels and/or lymphatic vessels in the stroma of breast cancer and the adjacent non-tumor tissues. Furthermore, the presence of Mortalin in breast cancer is also significantly associated with histological grade, LN metastasis and clinical stage, the three critical prognostic factors indicative of poor outcomes and cancer recurrence in breast cancer patients. Dundas et al. stated that in human colorectal adenocarcinoma, higher Mortalin expression correlated with poor patient survival [13]. In the present study, univariate survival analysis revealed that clinical stage, LN metastasis, Her2 expression level and Mortalin expression status are all significantly related with DFS and OS rates of patients with breast cancer (*P* < 0.05). Further multivariate survival analysis showed that Mortalin expression was one of the independent prognostic factors, along with clinical stage and Her2 status. Apparently, Mortalin may be a novel marker related to poor survival of breast cancer patients. Most importantly, breast cancer patients with overexpression of Mortalin concomitant with advanced clinical stages and lymph node metastasis had a lower survival rate than those with low Mortalin expression. Furthermore, Her2 positive was determined to be the important factor in predicting long-term DFS and OS of breast cancer patients. Chen et al. found that expression of Mortalin was notably higher in the SMMC 7721 (a liver-derived tumor cell line) than in a normal liver cell line [25]. Lu et al. showed that human HepG2 cells lacked mortalin-p53 interaction and were resistant to apoptosis, but cell apoptosis was significantly increased by Mortalin shRNA transfection [26]. Chen et al. also showed the conclusions that low expression of Mortalin was able to inhibit EMT, decrease tumor progression and lose the metastasis-inducing capability [24].

Despite of these interesting findings, larger sample size in randomized studies is needed to assess the potential value of Mortalin as candidate biomarker for breast cancer surveillance.

## Conclusions

Mortalin plays an important role in breast cancer progression; it might be a new attractive biomarker for prognostic evaluation and a molecular therapeutic target in patients with breast cancer.

## References

[1] Torre LA, Bray F, Siegel RL, Ferlay J, Lortet-Tieulent J, Jemal A (2015). Global cancer statistics, 2012. CA Cancer J Clin.

[2] Fan L, Strasser-Weippl K, Li JJ, St Louis J, Finkelstein DM, Yu KD, Chen WQ, Shao ZM, Goss PE (2014). Breast cancer in China. Lancet Oncol.

[3] Czarnecka AM, Campanella C, Zummo G, Cappello F (2006). Mortalin: when a close friend becomes a close enemy. Cancer Biol Ther.

[4] Wadhwa R, Takano S, Kaur K, Deocaris CC, Pereira-Smith OM, Reddel RR, Kaul SC (2006). Upregulation of mortalin/mthsp70/Grp75contributes to human carcinogenesis. Int J Cancer.

[5] Yang L, Liu X, Hao J, Yang Y, Zhao M, Zuo J, Liu W (2008). Glucose-regulated protein 75 suppresses apoptosis induced by glucose deprivation in PC12 cells through inhibition of Bax conformational change. Acta Biochem Biophys Sin (Shanghai).

[6] Mizukoshi E, Suzuki M, Misono T, Loupatov A, Munekata E, Kaul SC, Wadhwa R, Imamura T (2001). Cell-cycle dependent tyrosine phosphorylation on mortalin regulates its interaction with fibroblast growth factor-1. Biochem Biophys Res Commun.

[7] Wadhwa R, Takano S, Robert M, Yoshida A, Nomura H, Reddel RR, Mitsui Y, Kaul SC (1998). Inactivation of tumor suppressor p53 by mot-2, a hsp70 family member. J Biol Chem.

[8] Wadhwa R, Yaguchi T, Hasan MK, Taira K, Kaul SC (2003). Mortalin-MPD (mevalonate pyrophosphate decarboxylase) interactions and their role in control of cellular proliferation. Biochem Biophys Res Commun.

[9] Yi X, Luk JM, Lee NP, Peng J, Leng X, Guan XY, Lau GK, BerettaL FST (2008). Association of mortalin (HSPA9) with liver cancermetastasis and prediction for early tumor recurrence. Mol Cell Proteomics.

[10] Starenki D, Hong SK, Lloyd RV, Park JI (2015). Mortalin (GRP75/HSPA9) upregulation promotes survival and proliferation of medullary thyroid carcinoma cells. Oncogene.

[11] Li X, Oghi KA, Zhang J, Krones A, Bush KT, Glass CK, Nigam SK, Aggarwal AK, Maas R, Rose DW, Rosenfeld MG (2003). Eya protein phosphatase activity regulates Six1-Dach- Eya transcriptional effects in mammalian organogenesis. Nature.

[12] Elzagheid A, Kuopio T, IImen M, Collan Y (2002). Prognostication of invasive ductal breast cancer by quantificationof E-cadherin immunostaining: themethodology and clinical relevance. Histopathology.

[13] Dundas SR, Lawrie LC, Rooney PH, Murray GI (2005). Mortalin is over-expressed by colorectaladenocarcinomas and correlates with poor survival. J Pathol.

[14] Deocaris CC, Lu WJ, Kaul SC, Wadhwa R (2013). Druggability of mortalin for cancer and neuro-degenerativedisorders. Curr Pharm Des.

[15] Yang L, Guo W, Zhang Q, Li H, Liu X, Yang Y, Zuo J, Liu W (2011). Crosstalk between Raf/MEK/ERK and PI3K/AKT insuppression of Bax conformational change by Grp75 under glucose deprivation conditions. J Mol Biol.

[16] Wadhwa R, Ando H, Kawasaki H, Taira K, Kaul SC (2003). Targeting mortalin usingconventional and RNAhelicase-coupled hammerhead ribozymes. EMBO Rep.

[17] Yoo JY, Ryu J, Gao R, Yaguchi T, Kaul SC, Wadhwa R, Yun CO (2010). Tumor suppression by apoptotic and antiangiogenic effects of mortalin-targeting adeno-oncolytic virus. J Gene Med.

[18] Kaul SC, Aida S, Yaguchi T, Kaur K, Taira K, Wadhwa R (2005). Activation of wild type p53 function by its mortalin-binding cytoplasmically localizing carboxy-terminus peptides. J Biol Chem.

[19] Wadhwa R, Colgin L, Yaguchi T, Taira K, Reddel RR, Kaul SC (2002). Rhodacyanine dye MKT-077 inhibits in vitro telomerase assay but has no detectable effects on telomerase activity in vivo. Cancer Res.

[20] Wadhwa R, Sugihara T, Yoshida A, Nomura H, Reddel RR, Simpson R, Maruta H, Kaul SC (2000). Selective toxicity of MKT-077 to cancer cells is mediated by its binding to the hsp70 family protein mot-2 and reactivation of p53 function. Cancer Res.

[21] Widodo N, Kaur K, Shrestha BG, Takagi Y, Ishii T, Wadhwa R, Kaul SC (2007). Selective killing of cancer cells byleaf extract of Ashwagandha: identification of a tumor-inhibitory factor and the first molecular insights toits effect. Clin Cancer Res.

[22] Vaishnavi K, Saxena N, Shah N, Singh R, Manjunath K, Uthayakumar M, Kanaujia SP, Kaul SC, Sekar K, Wadhwa R (2012). Differential activities ofthe two closely related withanolides, Withaferin A and Withanone: bioinformatics and experimental evidences. PLoS One.

[23] Shin BK, Wang H, Yim AM, Le Naour F, Brichory F, Jang JH, ZhaoR PE, Tra J, Michael CW, Misek DE, Hanash SM (2003). Globalprofiling of the cell surface proteome of cancer cells uncovers anabundance of proteins with chaperone function. J Biol Chem.

[24] Chen J, Liu WB, Jia WD, Xu GL, Ma JL, Huang M, Deng YR, Li JS (2014). Overexpression of Mortalin in hepatocellular carcinoma and its relationship with angiogenesis and epithelial to mesenchymal transition. Int J Oncol.

[25] Chen X, Xu B, Li H, Yang L, Zuo J, Liu W, Liu C (2011). Expression of mortalin detected in human liver cancer by tissue microarrays. Anat Rec.

[26] Lu WJ, Lee NP, Kaul SC, Lan F, Poon RT, Wadhwa R, Luk JM (2011). Mortalin-p53 Interaction in cancer cells is stress dependent and constitutes a selective target for cancer therapy. Cell Death Differ.

